# Supplementary material for: Trapping Charge Mechanism in Hv1 Channels (CiHv1)
Source: Int J Mol Sci. 2023 Dec 28;25(1):426. doi: 10.3390/ijms25010426 (PMC10779229; doi:10.3390/ijms25010426)
Supplement: Supplementary file 1 [file ijms-25-00426-s001.zip › ijms-2757537-supplementary.pdf]

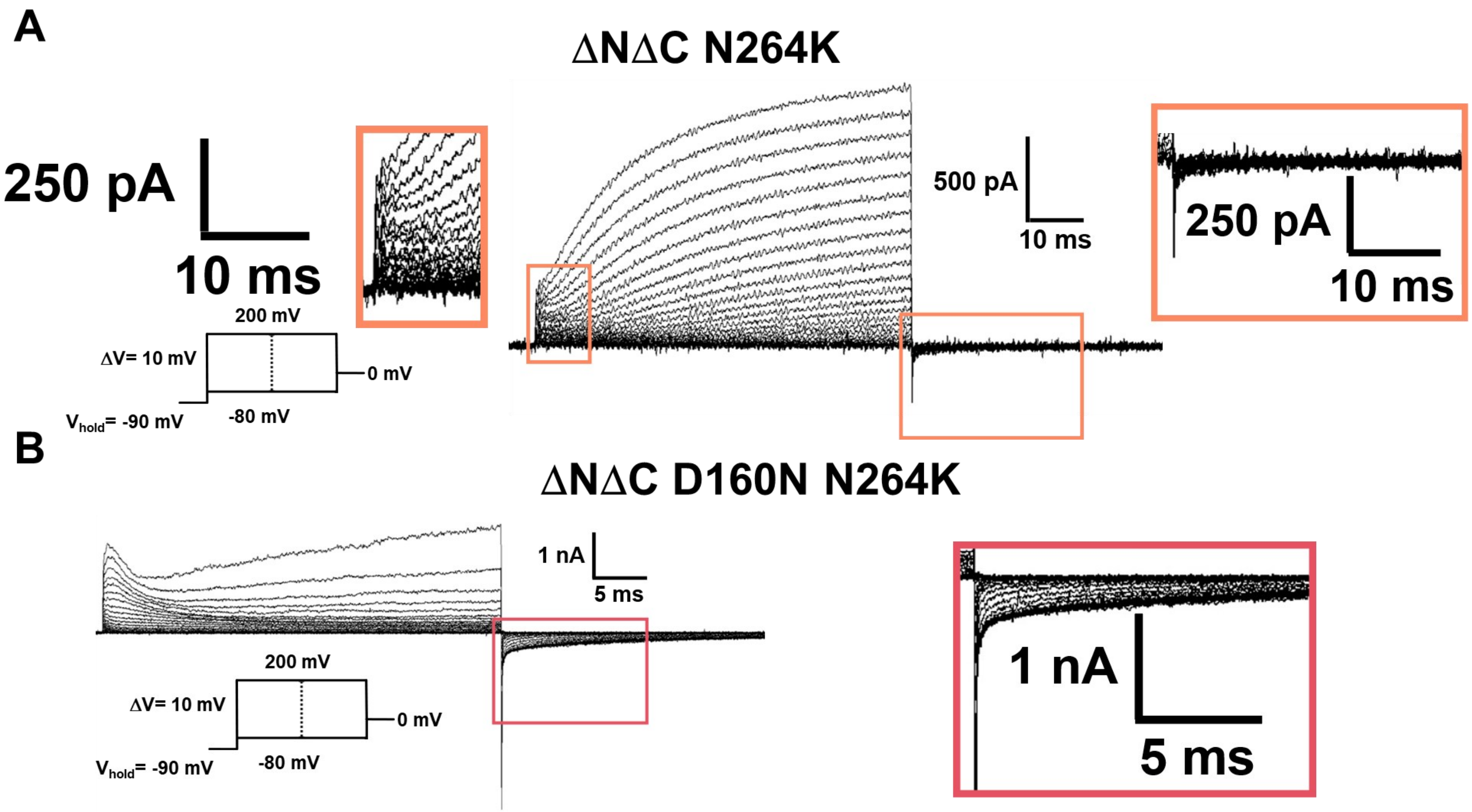

Figure S1. Positive charge in a lysine also promotes charge trapping. (A) The mutant ΔNΔC N264R produces a robust macroscopic current at the begining of the polarization, almost completely masking an OFF-gating current (left). On the other hand, in the OFF-gating current when repolarizing to 0 mv, it is possible to see a trapped OFF-gating current (right). **(B)** The double mutant ΔNΔC D160N exhibits a decreased macroscopic current that allows to see the channel's ON-gating current (left), while the OFF-gating current is also decreased (right).

Table S1. Parameters from Q(V) curves fitted to a two-state Boltzmann distribution for different mutation in the selectivity filter. Values obtained for the different mutants in the D160 $V_{0.5}$, $z\delta$ and ΔG positions are shown in Figures 3B and 3C. Data are shown as MEAN ± S.E.M.

	$V_{0.5}$ (mV)	$z\delta$	$\Delta\Delta G$ (kcal/mol)	N
**ΔNΔC D160N**	124.1 ± 6.2	1.14 ± 0.03	0 ± 0	7
**ΔNΔC D160Q**	146.3 ± 3.7	0.70 ± 0.04	2.14 ± 0.14	4
**ΔNΔC D160A**	189.6 ± 4.9	0.71 ± 0.01	4.18 ± 0.05	5
**ΔNΔC D160V**	202.5 ± 0.4	0.85 ± 0.02	1.24 ± 0.08	4
**ΔNΔC D160C**	202.7 ± 7.5	0.78 ± 0.03	0.019 ± 0.23	4
**ΔNΔC D160I**	195.8 ± 9.8	0.62 ± 0.06	0.66 ± 0.6	3

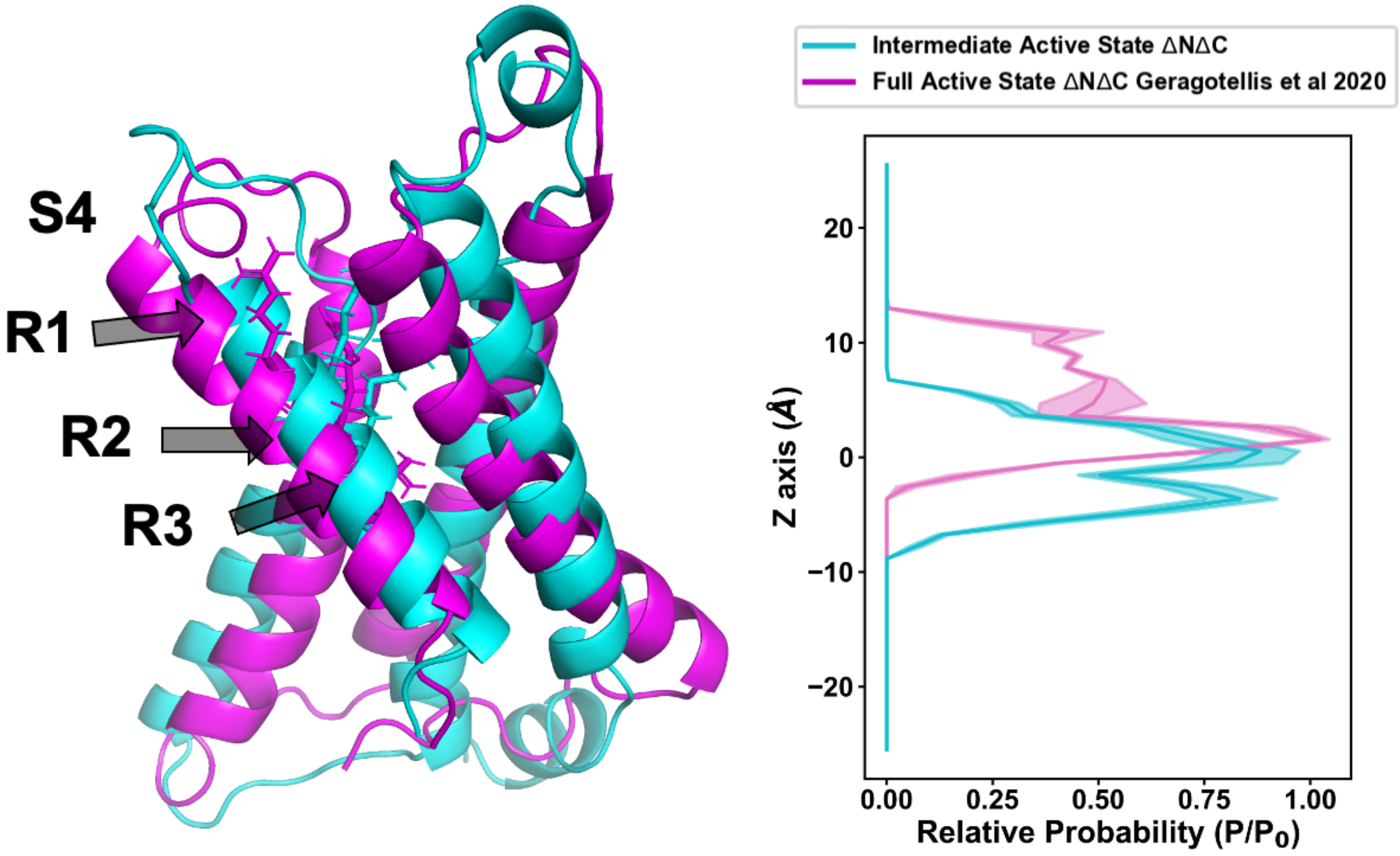

Figure S2 Comparison of structural active models between active *Ci*H$_v$1 models. (A) The S4 transmembrane segment where the voltage sensor is located is more displaced towards the extracellular side in the A_F model than in the A_I model. **(B)** The arginine density shows how the arginines of the A_F model tend to be positioned higher during the simulation than the arginines of the A_I model.

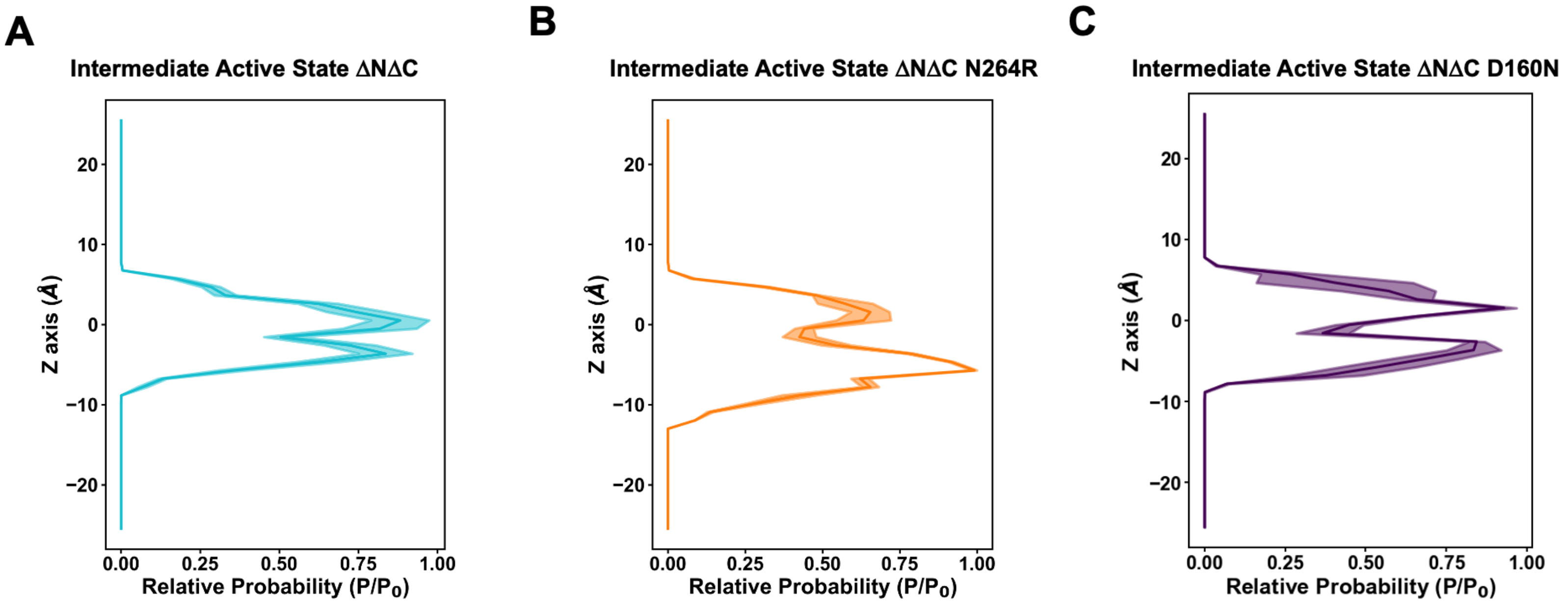

Figure S3. Intermediate Active State (A_I) for different mutants of *Ci*H_v1. The arginine density shows the probability of finding this amino acid in a given position for (A) ΔNΔC (B) ΔNΔC N264R (C) ΔNΔC D160N. For the ΔNΔC N264R mutant, the arginines are slightly shifted upward compared to the monomeric channel.

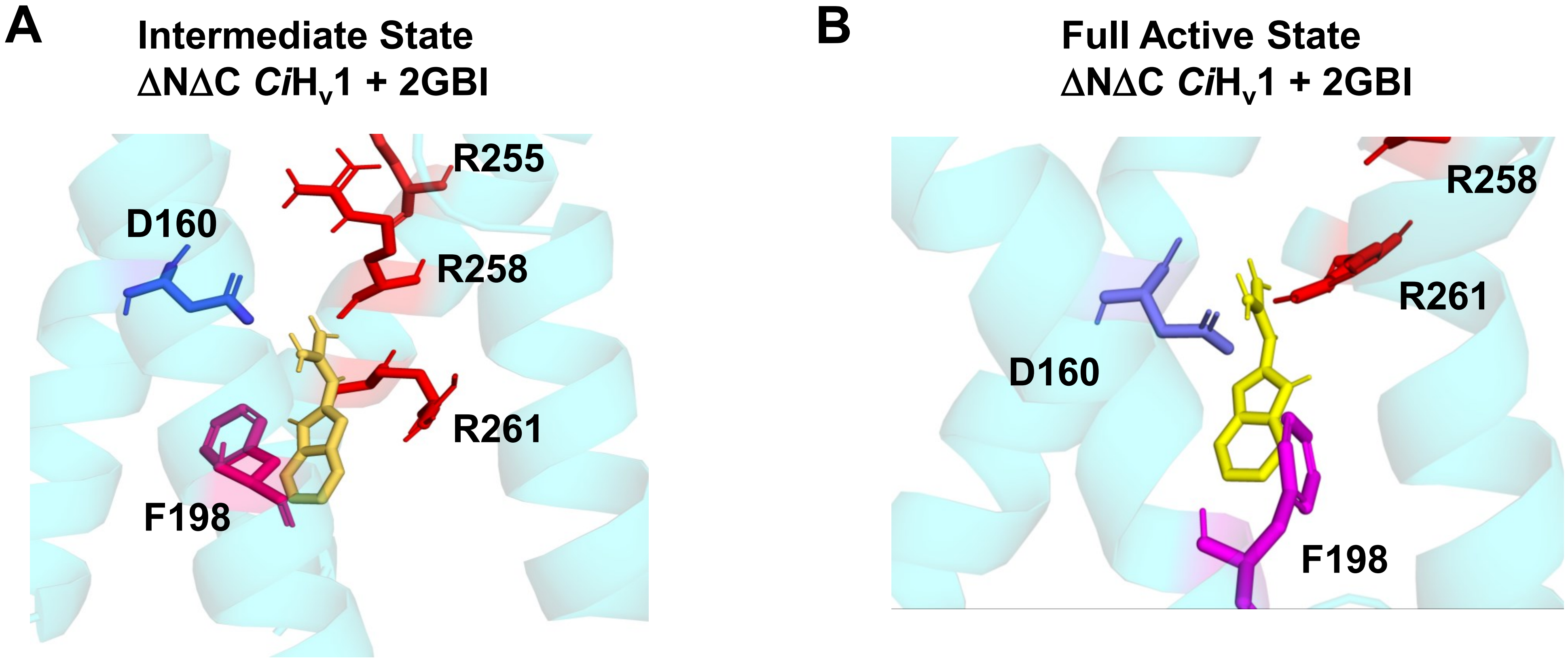

Figure S4. The molecular docking of 2GBI in two diferents active states. A) docked 2GBI for Intermediate Active State (A_I) and **B)** Full Active State (A_F) shown a similar interactions between selectivity filter D160 and guanidine group of 2GBI, F198 and benzoimidiazole ring of 2GBI and guanidine of 2GBI with R2 in Intermediate Active State and with R3 with Full Active State.

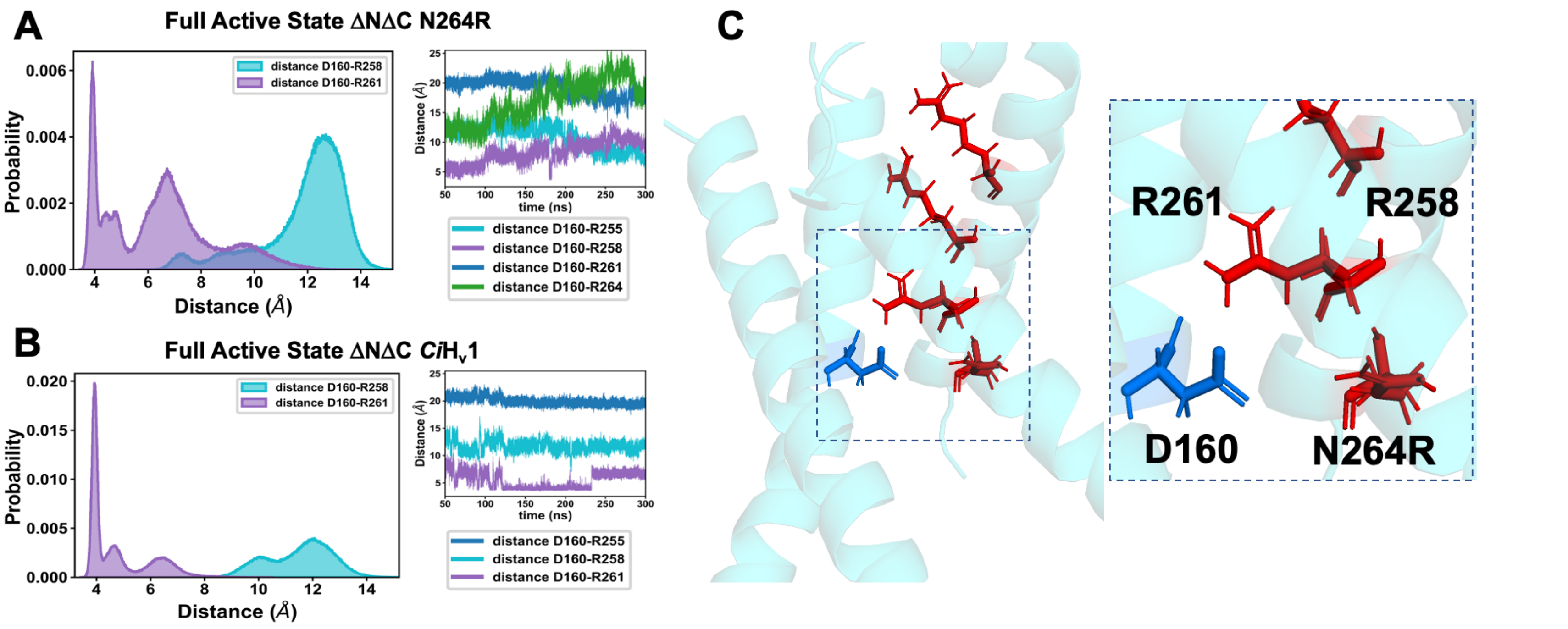

Figure S5. Full Active State ($A_{F'}$) model does not form stable salt-bridges over time. (A) and **(B)** Show the interaction of amino acids D160 and arginines 258 and 261 and how they fail to form salt-bridge interactions to stabilize the active structure in the ΔNΔC and ΔNΔC N264R respectively. **(C)** A representative structure shows how arginine 261 is the only arginine that can make salt-bridge interactions with position D160, while the other arginines are found in more distant positions for the ΔNΔC N264R mutant.

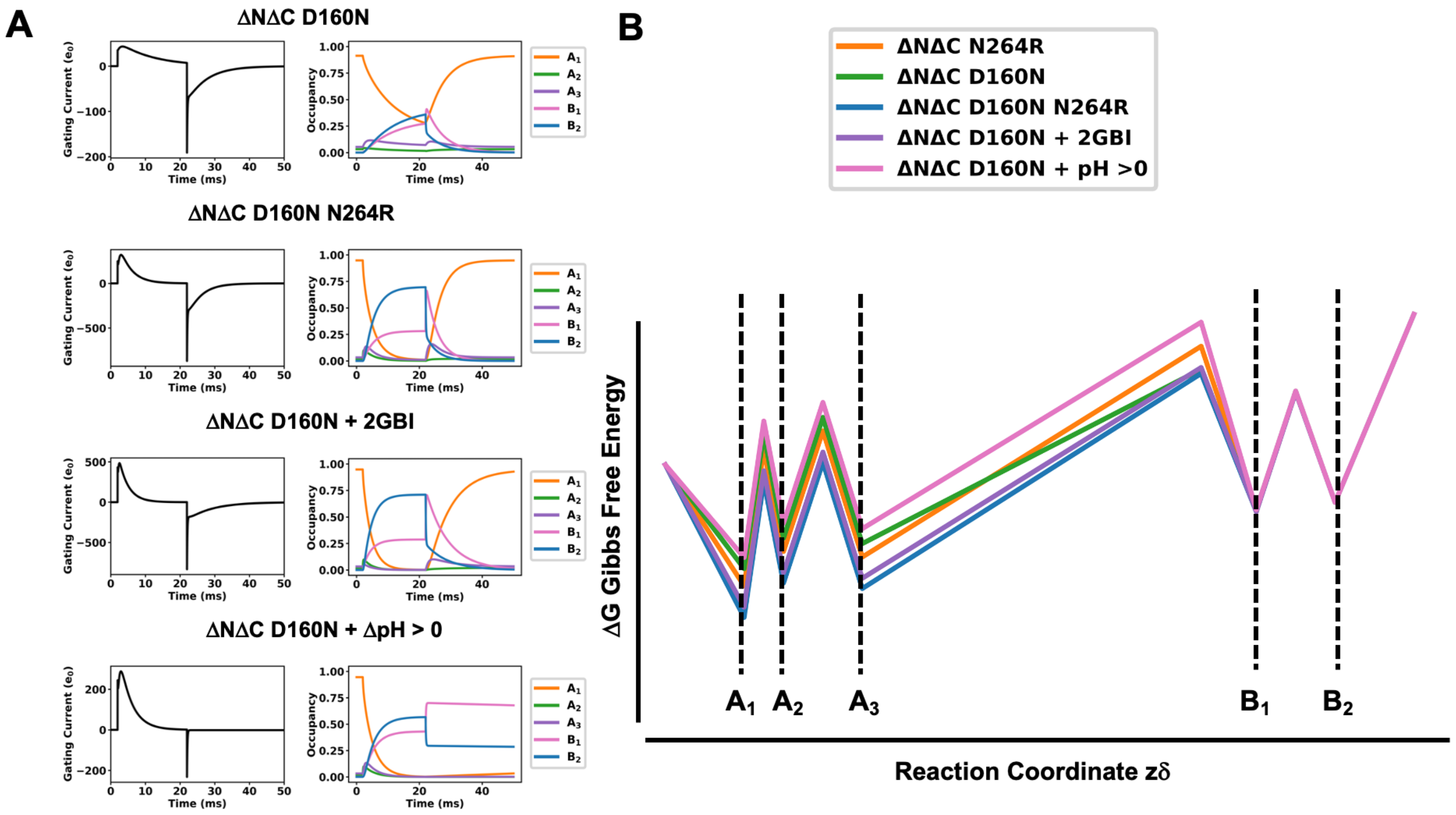

Figure S6. A Five-state model can reproduce the trapping mechanism in the different mutants. (A) With the five-state model it is possible to reproduce the shapes of the gating currents and in particular how they affect the OFF-gating current with the energy barrier separating states A_3 and B_1. On the right, the probability of the states as a function of time for each particular situation. **(B)** In general the five-state model can qualitatively predict charge trapping properties. Although the states of the smaller barriers may change, the transition between A3 and B1 is indispensable for charge trapping.

Table S3. Parameters of the five-state kinetic model of gating currents. Different values to the kinetic constants (α,β), and value of offset charge ($z\delta$) and charge fraction x for different conditions are shown in Figure S6.

ΔNΔC D160N

	A1 to A2	A2 to A3	A3 to B1	B1 to B2
α (ms)$^{-1}$	0.5	2.4	0.02	3
β (ms)$^{-1}$	12	1	0.2	5
$z\delta$	0.05	0.1	0.5	0.1
x	0.5	0.5	0.85	0.5

ΔNΔC D160N N264R

	A1 to A2	A2 to A3	A3 to B1	B1 to B2
α (ms)$^{-1}$	0.5	2.4	0.02	3
β (ms)$^{-1}$	12	1	0.2	5
$z\delta$	0.05	0.1	0.5	0.1
x	0.5	0.5	0.85	0.5

ΔNΔC D160N + 2GBI

	A1 to A2	A2 to A3	A3 to B1	B1 to B2
α (ms)$^{-1}$	0.5	2.4	0.002	3
β (ms)$^{-1}$	12	1	0.1	5
$z\delta$	0.2	0.1	1.2	0.1
x	0.5	0.5	0.85	0.5

ΔNΔC D160N + pH > 0

	A1 to A2	A2 to A3	A3 to B1	B1 to B2
α (ms)$^{-1}$	0.5	2.4	0.002	3
β (ms)$^{-1}$	12	1	0.001	5
$z\delta$	0.2	0.1	1.0	0.1
x	0.5	0.5	0.85	0.5

Table S2: Electrostatic interaction energy within the selectivity filter (D160) and arginines R2 and R3 of the voltage sensor in CiHv1.

**System**	**Pair Electrostatic Interaction Energy (Kcal/mol)**				**Total energy difference (Kcal/mol)**	
	**D-R2**	**D-R3**	**R2-R3**	**Total**	**$\Delta A_F \rightarrow A_I$***	**$\Delta\Delta\ A_F \rightarrow A_I$****
**ΔNΔC N264R A_I**	-2.77±0.09	-2.49±0.21	0.82±0.09	-4.44±0.25	3.03±0.56	2.62±1.11
**ΔNΔC N264R A_F**	-0.90±0.14	-1.80±0.47	1.29±0.10	-1.41±0.50		
**ΔNΔC WT A_I**	-2.30±0.41	-1.75±0.48	1.64±0.48	-2.41±0.79	0.41±0.96	
**ΔNΔC WT A_F**	-0.97±0.1	-2.35±0.49	1.32±0.22	-2.00±0.54		

*** Total Energy difference from the $\Delta\ A_F \rightarrow A_I$ transition.**
**** Total Energy difference of both systems (N264R→WT) and from the $\Delta A_F \rightarrow A_I$ transitions.**
